# Myeloid ACAT1/SOAT1: a novel regulator of dyslipidemia and retinal neovascularization

**DOI:** 10.1038/s44324-024-00046-x

**Published:** 2025-01-13

**Authors:** Syed A. H. Zaidi, Ruth B. Caldwell, Modesto A. Rojas

**Affiliations:** 1 Vascular Biology Center, Augusta University, Augusta, GA 30912 USA; 2 Vision Discovery Institute, Augusta University, Augusta, GA 30912 USA; 3 Department of Medicine, Augusta University, Augusta, GA 30912 USA; 4 Department of Cellular Biology and Anatomy, Augusta University, Augusta, GA 30912 USA; 5 Department of Pharmacology and Toxicology, Augusta University, Augusta, GA 30912 USA

**Keywords:** Metabolic disorders, Cell biology, Fat metabolism, Metabolic diseases

## Abstract

Pathological retinal neovascularization (RNV) is a major cause of vision loss and blindness during ischemic retinopathies. Our investigations in the mouse model of oxygen-induced retinopathy (OIR) demonstrate a novel mechanism of pathological RNV and neurovascular injury. We show that OIR-induced activation of macrophage/microglial cells, retinal inflammation, and pathological RNV are mediated by increases in cholesterol ester (CE) formation due to activation of the acyl-CoA: Cholesterol Acyltransferase 1/Sterol O-Acyltransferase 1 (ACAT1/SOAT1) enzyme.

## Introduction

Ischemic retinopathies, including diabetic retinopathy, retinopathy of prematurity, and retinal vein occlusion, are common causes of vision loss and blindness in developed countries worldwide^1–3^. Conventional therapies include repeated intravitreal injections of anti-VEGF (vascular endothelial growth factor), steroids, vitrectomy, or photocoagulation. All are associated with some risk of damaging side effects, including inflammation, retinal detachment, endophthalmitis, and vitreous hemorrhage. Rapid vascular regrowth may occur upon interruption of the VEGF blockade and its effectiveness is limited in some patients^4–7^. Steroid injections can induce increases in intraocular pressure or cataract formation and vitrectomy or photocoagulation can damage healthy cells. These limitations underscore the need for additional effective treatments that reverse RNV and promote physiological repair while avoiding the side effects of current therapeutic options.

In ischemic retinopathies hypoxia, oxidative stress, and inflammation are key factors underlying the retinal damage^8,9^. Activation of microglia and macrophages also plays a key role in ischemic retinopathy and both cell types are critically involved in the pathogenesis of pathological RNV^10–14^. Although these factors are all connected, the order of these events and their underlying mechanisms have not been well demonstrated. In this perspective review we will focus on the role of the cholesterol metabolizing enzyme ACAT1/SOAT1 and CE formation in retinal inflammation and pathological RNV in the mouse model of oxygen-induced retinopathy (OIR).

While dyslipidemia and cholesterol accumulation have been strongly implicated in promoting pathological NV in models of subretinal NV^15^, not much is known about role of cholesterol metabolism, activation of ACAT1/SOAT1, and CE formation in pathological RNV during ischemic retinopathy. More is known about the involvement of the ACAT1/SOAT1 pathway in other diseases. Increased ACAT1/SOAT1 expression/activity in activated macrophages has been implicated in atherosclerosis, Alzheimer’s disease, and cancer^16–18^. Epidemiological studies and clinical trials have shown a correlation between high levels of plasma cholesterol and diabetic retinopathy^19,20^. Alterations in cholesterol metabolism have also been shown to induce cholesterol accumulation and formation of hyperreflective cholesterol crystals that impair visual function in the diabetic retina^21^.

## Oxidative/nitrative stress, macrophage activation, inflammation, and pathological RNV

The mouse model of OIR has served as a key experimental tool for elucidating the mechanisms of pathological RNV. In this model, neonatal mice are maintained in hyperoxia from postnatal day 7 (P7) to P12, the time when the retinal vessels are forming, and then returned to normoxia. The hyperoxia environment induces obliteration of the immature vessels and prevents further microvascular development. Because the retinal neurons continue to develop normally during the hyperoxia treatment, the lack of the normal blood supply to the retinal tissue leads to relative hypoxia and promotes pathological vitreoretinal NV when the neonates are returned to normoxia on P12.

In models of OIR in vivo and hyperoxia-treated endothelial cells in vitro, oxidative/nitrative stress has been shown to play a key role in the hyperoxia-induced endothelial cell death^22,23^. There are several sources of oxidative/nitrative stress in the OIR model, including superoxide and peroxynitrite^24^. Superoxide formation due to activation of the NADPH oxidase NOX2 isoform together with nitric oxide formation due to increased expression of inducible nitric oxide synthase (iNOS) have been strongly implicated in retinal vascular injury during OIR and other models of ischemic retinopathy, including diabetic retinopathy and ischemia-reperfusion injury^25–28^. Superoxide and nitric oxide react to form the toxic product peroxynitrite, which damages the retinal cells as well as reducing the availability of nitric oxide needed for normal vascular and neuronal function^29^.

Protein synthesis pathways in the endoplasmic reticulum, Golgi apparatus, and nucleus as well as protein turnover pathways are all highly sensitive to ROS-related redox conditions^30^. Long chain unesterified free fatty acids along with some of their derivatives and metabolites can modify intracellular production of ROS^31^. Also, oxidative and nitrative stress mediate the activation of proinflammatory signaling pathways and promote induction of the M1-like proinflammatory macrophages during OIR^32,33^.

Studies in the OIR model have shown a key role of macrophage activation in this pathology. Depletion of macrophages during the relative hypoxia/RNV phase by intraperitoneal injections of clodronate-liposomes reduced retinal macrophage numbers by approximately 80% and significantly inhibited pathological RNV while improving physiological vascular repair^34^. In contrast, macrophage depletion during the hyperoxia phase did not affect the vaso-obliteration. These findings demonstrated that macrophage depletion markedly decreased OIR severity as well as reducing levels of angiogenic cytokines and extracellular matrix degradation^34,35^. Studies in the OIR model also have reported a population of M1-like proinflammatory microglia/macrophages localized to the areas of pathological RNV during the relative hypoxia/RNV phase along with activation of the NFκβ / STAT3 signaling pathway and increased expression of inflammatory cytokines including TNFα, IL6, and IL1β^35,36^.

In the OIR model, cells in the ischemic tissue promote pathological RNV by secreting VEGF and other angiogenic factors along with macrophage chemotactic protein 1 (MCP1), and macrophage colony stimulating factor (MCSF1)^14,34,37^. MCP1*/*CCL2 is a member of the C-C chemokine family and a potent chemotactic factor that attracts macrophages into the retina where they can release pro-angiogenic factors, promoting the growth of abnormal blood vessels. Once the macrophages are recruited, MCSF1 further activates them, enhancing their ability to produce inflammatory mediators and contribute to pathological angiogenesis^10^. It has been shown that inhibition of MCP1 decreases RNV^38^. MCSF1 secreted by macrophages, is required for the differentiation of resident macrophages and microglia during development^39^. Inhibition of MCSF1 selectively suppresses tumor angiogenesis and in contrast to VEGF blockade, MCSF1 inhibition does not promote rapid vascular regrowth^10,40^. Our studies in the OIR model have shown high levels of MCSF1 expression in the areas of pathological RNV^41^. Because macrophages can secrete MCSF1 as well as respond to it, our results suggest a proangiogenic role of MCSF1 released by activated macrophages as well as an additive effect of macrophage proliferation on pathological RNV.

## TREM1 (triggering receptor expressed on myeloid cells 1), inflammation, and pathological RNV

TREM1, a member of the immunoglobulin superfamily, has emerged as an important enhancer of inflammation. It activates neutrophils and monocytes/macrophages through the adapter protein DAP12^42^. TREM1 can be induced by several stimuli including hypoxia^43,44^ and may act as a stimulating factor for neuroinflammation. When activated in models of ischemic stroke or traumatic brain injury, TREM1 directly activates spleen tyrosine kinase (Syk) and its downstream signaling cascades in microglia^45,46^. This induces a proinflammatory microglial phenotype that can lead to neurotoxicity. Our studies in the mouse model of OIR have shown that hypoxia robustly increases expression of TREM1 together with MCSF1^41^. We also have found that TREM1 is localized to immune cells positive for isolectin B4, Iba1, CD45, and to MCSF1 positive immune cells in areas of pathological RNV. Moreover, specific inhibition of TREM1 blocked the pathological RNV and improved physiological vascular repair. This was the first study to show that TREM1 plays a major role in the progression of RNV in mice with OIR and suggested that activated microglia/macrophages are the main source of TREM1 expression^41^.

## Dyslipidemia and TREM1 activation

Lipids are a major building block in all living cells. They are involved in a wide variety of structural and functional cellular processes. More than 50% of the brain’s dry weight is composed of lipids, which are found as phospholipids, sphingolipids, glycerol lipids, fatty acids, and sterols, with phospholipids accounting for ~ 50% of total lipid content^47^. Functionally, lipids are key components of cell membranes including neuronal synapses and myelin sheaths. Within cell membranes they can serve to transduce signals to regulate a range of biological processes and in some circumstances act as bioenergetic fuels^48^. In these processes cholesterol esterification is a physiological mechanism used to store and transfer cholesterol in order to maintain cellular homeostasis and avoid cellular toxicity due to excess levels of unesterified cholesterol (also called free cholesterol)^49^.

Studies in models of atherosclerosis have demonstrated a synergistic interaction between dyslipidemia and activation of TREM1 in myeloid cells that leads to increases in production of proinflammatory cytokines^50^. Furthermore, hypercholesterolemia was found to induce TREM1 expression in myeloid cells and to subsequently increase myeloid cell differentiation, thereby increasing monocyte development and promoting proatherogenic cytokine production and foam cell formation. Pharmacological blockade of TREM1 in the *db/db* and high fat diet/streptozotocin-induced diabetic mouse models of type 2 diabetes has been shown to inhibit accumulation of lipid droplets, reduce inflammatory damage to hippocampal neurons, and improve cognitive functions^51^.

## ACAT1/SOAT1 (Acyl-coenzyme A: Cholesterol Acyltransferase 1/ Sterol O-Acyltransferase 1) and RNV

ACAT/SOAT are microsomal membrane-bound enzymes that are localized to the endoplasmic reticulum. They use cholesterol and long chain fatty acid as substrates to produce cholesterol esters (CE)^52^. ACAT/SOAT enzymes are expressed in many tissues including brain, retina, liver, and other tissues, where they are involved in formation and accumulation of CE^16,53^. In mammals, ACAT/SOAT exists as two isoforms: ACAT1/SOAT1 and ACAT2/SOAT2. Most tissues express ACAT1/SOAT1 as the major isoform and ACAT2/SOAT2 as a minor isoform^54^. Both isoforms are allosterically activated by their substrate (cholesterol or oxysterols). Cholesterol activates ACAT1/SOAT1, induces its expression allosterically, and is more effective than other sterols in this effect.

High levels of ACAT1/SOAT1 activity have been shown in a variety of disease conditions, including cancer, Alzheimer’s disease, and cardiovascular disease^55–57^. Studies using macrophages have shown that internalization of low density lipoprotein (LDL) cholesterol by the LDL receptor and the resulting increases in intracellular cholesterol levels increase ACAT1/SOAT1 activity which leads to increases in CE formation^58^. Our studies in the OIR mouse model have shown that increased expression of inflammatory mediators and pathological RNV are associated with increased expression of LDLR, increased accumulation of neutral lipids, and elevated levels of CE in areas of RNV along with high expression of ACAT1/SOAT1^59^. ACAT2/SOAT2 expression was unchanged during OIR. The ACAT1/SOAT1 protein was colocalized with TREM1, MCSF1, and markers of macrophage and microglial cells in areas of pathological RNV. Consistent with these findings, recent lipidomic analysis showed marked elevation of cholesterol esterification, lipid droplet formation, reversed cholesterol transport, and increased concentration of n-9 fatty acids along with increased expression of ACAT1/SOAT1 in the OIR retinas at P17 as compared with the room air controls^60^.

Our studies further showed that deletion of the LDL receptor completely blocks pathological RNV which indicates a critical role of LDL cholesterol uptake in the pathology^59^. Moreover, treatment with the specific inhibitor of ACAT1/SOAT1 K604 during the relative hypoxia phase of OIR significantly inhibited the increases in inflammatory mediators and limited the pathological RNV while reducing the avascular area, indicating involvement of CE formation in the pathology. Also, OIR-induced increases in retinal ACAT1/SOAT1 expression/activity together with high levels of CE formation and accumulation of neutral lipids were associated with increases in the expression of VEGF. In contrast with the effects of K604 treatment in reducing signs of inflammation and limiting the pathological RNV, VEGF expression was not affected^59^. Our studies using microglial cells exposed to oxygen-glucose deprivation (OGD) under in vitro conditions further showed marked increases in expression of ACAT1/SOAT1, TREM1, and MCSF along with significant increases in expression of VEGF^59^. Treatment with the K604 ACAT1/SOAT1 inhibitor blocked each of these effects except for the increase in VEGF expression.

**Fig. 1:**
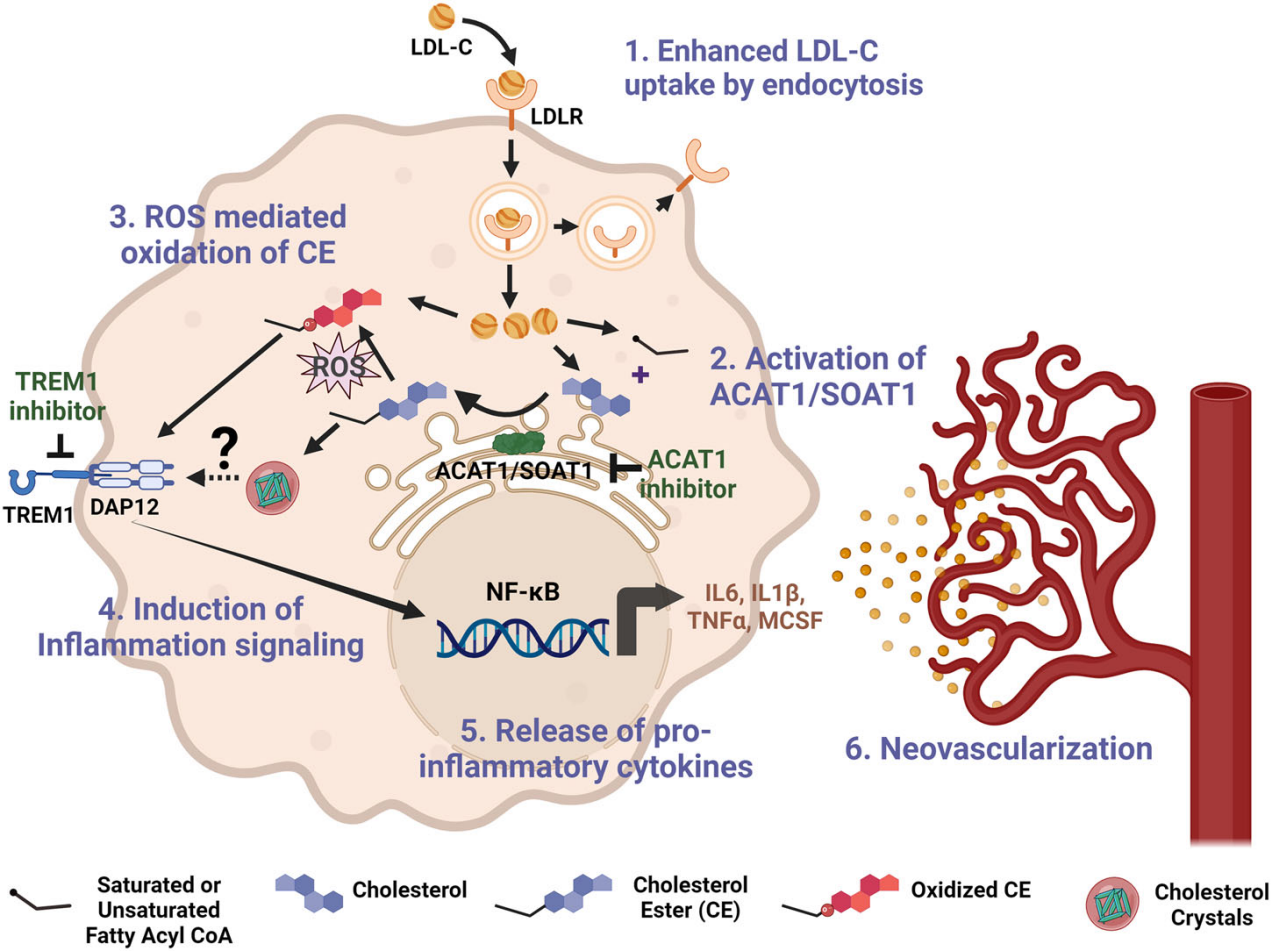
A simplified schematic representation of steps linking dyslipidemia with macrophage/microglia induced RNV. 1) Enhanced LDL-cholesterol uptake: Under hypoxic/ischemic conditions, macrophages/microglia show increased expression of LDL-receptor (LDLR) and increased internalization of LDL-cholesterol (LDL-C). 2) Activation of ACAT1/SOAT1: Hypoxic/ischemic conditions promote increased cholesterol uptake along with increased ACAT1/SOAT1 activity which increases CE formation. 3) ROS mediated oxidation of CE: Ischemia induces ROS formation, which leads to CE oxidation (Ox-CE) via activation of radical and nonradical modification pathways^69^. 4) Induction of Inflammation signaling: Ox-CE can activate TREM1 through TLR4^59,68^. TREM1 induces inflammation by activating the DAP12/Syk (spleen tyrosine kinase) signaling cascade. TREM1 inhibition decreases levels of inflammatory cytokines^65^. 5) Release of proinflammatory cytokines: The nuclear factor-κB (NFκβ) signaling pathway is induced by TREM1, which leads to the production of proinflammatory cytokines such as IL6, IL1β, TNFα, MCSF^41,70^ 6) Retinal neovascularization: Activated macrophages/microglia play a key role in regulating angiogenesis and are critically involved in the pathogenesis of RNV. Created in BioRender. Zaidi, S. (2025) BioRender.com/c38e697.

These data suggest that the increase in VEGF expression in the models of OIR or OGD is independent of ACAT1/SOAT1 activity. Although intravitreal injections of anti-VEGF are often used to treat RNV, potential side effects can limit its use^4^. VEGF is a critical survival factor required for physiological vascular growth and repair^61,62^. In our OIR study we also observed increased numbers of tip cells sprouting into the avascular area of retinas of OIR mice treated with K604 and a reduction in the avascular area as compared with the vehicle treated ischemic retinas. This suggests that inhibition of ACAT1/SOAT1 can limit pathological RNV while preserving normal VEGF expression and thereby promoting physiological vascular repair. This strategy may offer an effective therapy for pathological neovascularization. Figure 1 illustrates a suggested signaling pathway involved in ACAT1/SOAT1-induced RNV.

## Dyslipidemia, macrophage activation, and inflammation

Our studies have implicated ACAT1/SOAT1 activity and CE formation in the OIR-induced increases in expression of LDLR and TREM1 along with MCSF1 as well as inflammatory cytokines [59]. We and others have shown that TNFα is involved in pathological RNV, breakdown of the blood retinal barrier, and ACAT1/SOAT1 expression^59,63,64^. The mechanism of hyperlipidemia-induced macrophage activation and the role of TREM1 in the process are not fully understood. While several endogenous ligands for TREM1 have been identified, the mechanism of their activation is still not clear^65^.

One possible mechanism may involve activation of the toll like receptor 4 (TLR4)-Syk pathway. In a macrophage model of dyslipidemia, activation of TLR4 has been shown to play a key role in ROS formation. Stimulation of peritoneal macrophages with minimally modified LDL cholesterol (mmLDL) has been shown to promote significant increases in NOX2-derived ROS leading to increases in expression of inflammatory cytokines^66^. This effect was completely blocked in TLR4 knockout mice. In contrast, macrophages from MyD88 (myeloid differentiation primary response protein) knockout mice showed a normal ROS response. This suggests that mmLDL cholesterol-induced NOX2 derived ROS production in macrophages depends on the presence of TLR4 but not MyD88. Also, LDL cholesterol-induced activation of TLR4 in macrophages has been shown to involve TLR4-mediated Syk activation and downstream signaling. J774 macrophages stimulated with LDL cholesterol showed increased coimmunoprecipitation of Syk with TLR4^66^. In a recent study using microglial N9 cells, it was found that blocking ACAT1 with K604 significantly reduced the proinflammatory responses induced by LPS. K604 decreased the total TLR4 protein content by promoting TLR4 endocytosis, which enhanced the trafficking of TLR4 to the lysosomes for degradation. This suppression of TLR4 resulted in the inhibition of its proinflammatory signaling cascade in response to LPS^67^. The mechanism of this effect is as yet unknown, but may involve ACAT1/SOAT1 inhibitor-induced alterations in TLR4 membrane microdomains that cause it to undergo an increase in caveolae-mediated endocytosis.

Another likely mechanism is that oxidized CE could be involved. Studies have shown that oxidized cholesteryl arachidonate with bicyclic endoperoxide and hydroperoxide groups (BEP-CE) works as a specific oxidized CE to activate macrophages in a TREM2/MD2 (myeloid differentiation factor 2)-dependent manner^68^. MD2 is a TREM2 binding protein that dimerizes with TLR4 and is required for TLR4 activation. BEP-CE induces TLR4/MD2 binding and TLR4 dimerization which promotes phosphorylation of SyK, ERK1/2, JNK and c-Jun, cell spreading, and uptake of dextran and native LDL cholesterol by macrophages^68^. Taken together, these results suggest involvement of different sources of free radicals in the process of beta oxidation of LDL cholesterol which converts CEs into active forms capable of activating other enzymes or inducing the expression on other proteins. More studies are needed to fully elucidate the TREM1/TLR4/ SyK signaling pathway in ischemic retinopathy.

## Conclusion

We have demonstrated a novel strategy to limit pathological RNV and promote physiological repair in the mouse OIR model by inhibiting ACAT1/SOAT1 activity and thereby preventing CE formation and blocking activation of TREM1. The mechanism behind hypoxia-induced inflammation and macrophage/microglia activation is not yet clear. Our studies in the OIR model have shown that LDLR is induced by hypoxia and that LDLR deletion completely blocks intravitreal RNV^59^ which strongly supports the role of LDL cholesterol uptake in the pathology. In the OIR model, inhibition of ACAT1/SOAT1 significantly reduces inflammation and inhibits pathological RNV while improving vascular repair without altering VEGF expression. Anti-VEGF therapy during ischemic retinopathy is associated with some risks of neurovascular dysfunction since VEGF is a survival factor required for retinal neuronal function and vascular repair, especially during OIR. Collectively, dysregulation of retinal lipid metabolism in ischemic retinopathy can impact a variety of factors involved in inflammation and neurovascular damage and therefore visual functions. In summary targeting ACAT1/SOAT1 activity offers a novel strategy for treatment of ischemic retinopathies and other diseases that involve pathological RNV.

### Limitations and future directions

Under normal conditions ACAT1/SOAT1 is important for a variety of homeostatic functions, particularly in relation to immune function. However research in models of Alzheimer’s and cardiovascular disease has shown that excessive CE formation leads to the activation of myeloid cells and subsequently to cellular damage and that this can be blocked by inhibiting ACAT1/SOAT1 activity^55–57^. While the mechanism of this protective action is not fully established, results of studies using a model of LPS-induced neuroinflammation suggest that this protective effect involves alterations in the caveolae/lipid raft region of the plasma membrane^67^.

The above mentioned work in Alzheimer’s and cardiovascular disease models has shown that blockade of ACAT1/SOAT1 can limit disease pathology without impairing normal physiological functions. However considering that neonatal mice are undergoing normal development, we monitored body weight, motility, behavior, and plasma levels of cytokines and lipid profiles after treatment with the ACAT1/SOAT1 inhibitor or vehicle control. Those analyses showed no adverse effects with the inhibitor treatment. Our data further showed that inhibitor treatment over a period of 10 days inhibits pathological RNV and promotes physiological vascular repair while limiting vision loss tested after the mice mature. In comparison with anti-VEGF, the ACAT1/SOAT1 inhibitor treatment should be safer since blocking VEGF affects all cells that require it for their normal function including neurons as well as vascular endothelial cells.

In our study we did not identify subtypes of CEs. Their identification will be key to defining more clearly the role of CE in this pathology. To access this, correlating a lipidomic analysis with studies of the expression of ACAT1/SOAT1, TREM1, MCSF1, VEGF and inflammatory cytokines should be a priority. Our further studies will explore the known markers of retinal neuronal damage, including vascular permeability, leukocyte adhesion, acellular capillary formation, retinal inflammation, and oxidative stress in relation to the tissue damage induced by ACAT1/SOAT1 in models of diabetic retinopathy (i.e., Akita mice, STZ-induced diabetic mice, *db/db* mice). Studies using strategies for deletion of ACAT1/SOAT1 in macrophage and retinal microglial cells will give us a better understanding of the underlying mechanisms.

## Data Availability

No datasets were generated or analysed during the current study.

## References

[1] Laouri, M., Chen, E., Looman, M. & Gallagher, M. The burden of disease of retinal vein occlusion: review of the literature. *Eye (Lond.)* **25**, 981–988, 10.1038/eye.2011.92 (2011). 21546916 10.1038/eye.2011.92 PMC3178209

[2] Mutlu, F. M. & Sarici, S. U. Treatment of retinopathy of prematurity: a review of conventional and promising new therapeutic options. *Int. J. Ophthalmol.* **6**, 228–236, 10.3980/j.issn.2222-3959.2013.02.23 (2013). 23641347 10.3980/j.issn.2222-3959.2013.02.23 PMC3633766

[3] Kropp, M. et al. Diabetic retinopathy as the leading cause of blindness and early predictor of cascading complications-risks and mitigation. *EPMA J.* **14**, 21–42, 10.1007/s13167-023-00314-8 (2023). 36866156 10.1007/s13167-023-00314-8 PMC9971534

[4] Pieramici, D. J. & Rabena, M. D. Anti-VEGF therapy: comparison of current and future agents. *Eye (Lond.)* **22**, 1330–1336, 10.1038/eye.2008.88 (2008). 18497829 10.1038/eye.2008.88

[5] Maharaj, A. S. et al. VEGF and TGF-beta are required for the maintenance of the choroid plexus and ependyma. *J. Exp. Med.* **205**, 491–501, 10.1084/jem.20072041 (2008). 18268040 10.1084/jem.20072041 PMC2271023

[6] Verheul, H. M. & Pinedo, H. M. Possible molecular mechanisms involved in the toxicity of angiogenesis inhibition. *Nat. Rev. Cancer* **7**, 475–485, 10.1038/nrc2152 (2007). 17522716 10.1038/nrc2152

[7] Mancuso, M. R. et al. Rapid vascular regrowth in tumors after reversal of VEGF inhibition. *J. Clin. Invest.* **116**, 2610–2621, 10.1172/JCI24612 (2006). 17016557 10.1172/JCI24612 PMC1578604

[8] Graziosi, A., et al. Oxidative stress markers and the retinopathy of prematurity. *J. Clin. Med*. 9, 2711 (2020). 10.3390/jcm9092711. 10.3390/jcm9092711 PMC7563779 32825796

[9] Tang, J. & Kern, T. S. Inflammation in diabetic retinopathy. *Prog. Retin. Eye Res.* **30**, 343–358, 10.1016/j.preteyeres.2011.05.002 (2011). 21635964 10.1016/j.preteyeres.2011.05.002 PMC3433044

[10] Kubota, Y. et al. M-CSF inhibition selectively targets pathological angiogenesis and lymphangiogenesis. *J. Exp. Med.* **206**, 1089–1102, 10.1084/jem.20081605 (2009). 19398755 10.1084/jem.20081605 PMC2715025

[11] Checchin, D. et al. Potential role of microglia in retinal blood vessel formation. *Invest. Ophthalmol. Vis. Sci.* **47**, 3595–3602, 10.1167/iovs.05-1522 (2006). 16877434 10.1167/iovs.05-1522

[12] Espinosa-Heidmann, D. G. et al. Macrophage depletion diminishes lesion size and severity in experimental choroidal neovascularization. *Invest. Ophthalmol. Vis. Sci.* **44**, 3586–3592, 10.1167/iovs.03-0038 (2003). 12882811 10.1167/iovs.03-0038

[13] Sakurai, E. et al. Macrophage depletion inhibits experimental choroidal neovascularization. *Invest. Ophthalmol. Vis. Sci.* **44**, 3578–3585, 10.1167/iovs.03-0097 (2003). 12882810 10.1167/iovs.03-0097

[14] Zhou, Y. D. et al. Diverse roles of macrophages in intraocular neovascular diseases: a review. *Int. J. Ophthalmol.* **10**, 1902–1908, 10.18240/ijo.2017.12.18 (2017). 29259911 10.18240/ijo.2017.12.18 PMC5733520

[15] Pikuleva, I. A. & Curcio, C. A. Cholesterol in the retina: the best is yet to come. *Prog. Retin Eye Res.* **41**, 64–89, 10.1016/j.preteyeres.2014.03.002 (2014). 24704580 10.1016/j.preteyeres.2014.03.002 PMC4058366

[16] Chang, T. Y., Li, B. L., Chang, C. C. & Urano, Y. Acyl-coenzyme A:cholesterol acyltransferases. *Am. J. Physiol. Endocrinol. Metab.* **297**, E1–E9, 10.1152/ajpendo.90926.2008 (2009). 19141679 10.1152/ajpendo.90926.2008 PMC2711667

[17] Shibuya, Y., Chang, C. C. & Chang, T. Y. ACAT1/SOAT1 as a therapeutic target for Alzheimer’s disease. *Future Med. Chem.* **7**, 2451–2467, 10.4155/fmc.15.161 (2015). 26669800 10.4155/fmc.15.161 PMC4976859

[18] Zabielska, J., Sledzinski, T. & Stelmanska, E. Acyl-coenzyme a: cholesterol acyltransferase inhibition in cancer treatment. *Anticancer Res* **39**, 3385–3394, 10.21873/anticanres.13482 (2019). 31262860 10.21873/anticanres.13482

[19] Busik, J. V. Lipid metabolism dysregulation in diabetic retinopathy. *J. Lipid Res* **62**, 100017, 10.1194/jlr.TR120000981 (2021). 33581416 10.1194/jlr.TR120000981 PMC7892987

[20] Chou, Y., Ma, J., Su, X. & Zhong, Y. Emerging insights into the relationship between hyperlipidemia and the risk of diabetic retinopathy. *Lipids Health Dis.* **19**, 241, 10.1186/s12944-020-01415-3 (2020). 33213461 10.1186/s12944-020-01415-3 PMC7677820

[21] Jenkins, A. J., Grant, M. B. & Busik, J. V. Lipids, hyperreflective crystalline deposits and diabetic retinopathy: potential systemic and retinal-specific effect of lipid-lowering therapies. *Diabetologia* **65**, 587–603, 10.1007/s00125-022-05655-z (2022). 35149880 10.1007/s00125-022-05655-z PMC9377536

[22] Brooks, S. E. et al. Reduced severity of oxygen-induced retinopathy in eNOS-deficient mice. *Invest. Ophthalmol. Vis. Sci.* **42**, 222–228 (2001). 11133872

[23] Gu, X. L. et al. Hyperoxia induces retinal vascular endothelial cell apoptosis through formation of peroxynitrite. *Am. J. Physiol.-Cell Ph* **285**, C546–C554, 10.1152/ajpcell.00424.2002 (2003). 10.1152/ajpcell.00424.2002 12736139

[24] Suwanpradid, J. et al. Arginase 2 deficiency prevents oxidative stress and limits hyperoxia-induced retinal vascular degeneration. *PLoS One* **9**, e110604, 10.1371/journal.pone.0110604 (2014). 25375125 10.1371/journal.pone.0110604 PMC4222858

[25] Chan, E. C. et al. Involvement of Nox2 NADPH oxidase in retinal neovascularization. *Invest Ophthalmol. Vis. Sci.* **54**, 7061–7067, 10.1167/iovs.13-12883 (2013). 24106122 10.1167/iovs.13-12883

[26] Rojas, M. et al. Requirement of NOX2 expression in both retina and bone marrow for diabetes-induced retinal vascular injury. *PLoS One* **8**, e84357, 10.1371/journal.pone.0084357 (2013). 24358357 10.1371/journal.pone.0084357 PMC3866146

[27] Al-Shabrawey, M. et al. Role of NADPH oxidase in retinal vascular inflammation. *Invest Ophthalmol. Vis. Sci.* **49**, 3239–3244, 10.1167/iovs.08-1755 (2008). 18378574 10.1167/iovs.08-1755 PMC3798055

[28] Yokota, H. et al. Neuroprotection from retinal ischemia/reperfusion injury by NOX2 NADPH oxidase deletion. *Invest Ophthalmol. Vis. Sci.* **52**, 8123–8131, 10.1167/iovs.11-8318 (2011). 21917939 10.1167/iovs.11-8318 PMC3208002

[29] Narayanan, S. P. et al. Arginase in retinopathy. *Prog. Retin Eye Res* **36**, 260–280, 10.1016/j.preteyeres.2013.06.002 (2013). 23830845 10.1016/j.preteyeres.2013.06.002 PMC3759622

[30] Moldovan, L. & Moldovan, N. I. Oxygen free radicals and redox biology of organelles. *Histochem Cell Biol.* **122**, 395–412, 10.1007/s00418-004-0676-y (2004). 15452718 10.1007/s00418-004-0676-y

[31] Schonfeld, P. & Wojtczak, L. Fatty acids as modulators of the cellular production of reactive oxygen species. *Free Radic. Biol. Med* **45**, 231–241, 10.1016/j.freeradbiomed.2008.04.029 (2008). 18482593 10.1016/j.freeradbiomed.2008.04.029

[32] Perez, S., Rius-Perez, S. Macrophage polarization and reprogramming in acute inflammation: a redox perspective. *Antioxidants (Basel)*. **11**, 1394 10.3390/antiox11071394 (2022). 10.3390/antiox11071394 PMC9311967 35883885

[33] Fouda, A. Y. et al. Targeting proliferative retinopathy: Arginase 1 limits vitreoretinal neovascularization and promotes angiogenic repair. *Cell Death Dis.* **13**, 745, 10.1038/s41419-022-05196-8 (2022). 36038541 10.1038/s41419-022-05196-8 PMC9424300

[34] Gao, X. et al. Macrophages promote vasculogenesis of retinal neovascularization in an oxygen-induced retinopathy model in mice. *Cell Tissue Res.* **364**, 599–610, 10.1007/s00441-015-2353-y (2016). 26841878 10.1007/s00441-015-2353-y

[35] Li, J. et al. The phase changes of M1/M2 phenotype of microglia/macrophage following oxygen-induced retinopathy in mice. *Inflamm. Res*. **70**, 183–192, 10.1007/s00011-020-01427-w (2021). 33386422 10.1007/s00011-020-01427-w

[36] Mechoulam, H. & Pierce, E. A. Expression and activation of STAT3 in ischemia-induced retinopathy. *Invest. Ophthalmol. Vis. Sci.* **46**, 4409–4416, 10.1167/iovs.05-0632 (2005). 16303927 10.1167/iovs.05-0632

[37] Shahror, R. A. et al. Role of myeloid cells in ischemic retinopathies: recent advances and unanswered questions. *J. Neuroinflammation* **21**, 65, 10.1186/s12974-024-03058-y (2024). 38454477 10.1186/s12974-024-03058-y PMC10918977

[38] Yoshida, S. et al. Role of MCP-1 and MIP-1alpha in retinal neovascularization during postischemic inflammation in a mouse model of retinal neovascularization. *J. Leukoc. Biol.* **73**, 137–144, 10.1189/jlb.0302117 (2003). 12525571 10.1189/jlb.0302117

[39] Cecchini, M. G. et al. Role of colony stimulating factor-1 in the establishment and regulation of tissue macrophages during postnatal development of the mouse. *Development* **120**, 1357–1372, 10.1242/dev.120.6.1357 (1994). 8050349 10.1242/dev.120.6.1357

[40] Aharinejad, S. et al. Colony-stimulating factor-1 blockade by antisense oligonucleotides and small interfering RNAs suppresses growth of human mammary tumor xenografts in mice. *Cancer Res* **64**, 5378–5384, 10.1158/0008-5472.CAN-04-0961 (2004). 15289345 10.1158/0008-5472.CAN-04-0961

[41] Rojas, M. A., Shen, Z. T., Caldwell, R. B. & Sigalov, A. B. Blockade of TREM-1 prevents vitreoretinal neovascularization in mice with oxygen-induced retinopathy. *Biochim Biophys. Acta Mol. Basis Dis.* **1864**, 2761–2768, 10.1016/j.bbadis.2018.05.001 (2018). 29730341 10.1016/j.bbadis.2018.05.001 PMC6488934

[42] Colonna, M. & Facchetti, F. TREM-1 (triggering receptor expressed on myeloid cells): a new player in acute inflammatory responses. *J. Infect. Dis.* **187**, S397–S401, 10.1086/374754 (2003). 12792857 10.1086/374754

[43] Mishra, K. P., Jain, S., Ganju, L. & Singh, S. B. Hypoxic stress induced TREM-1 and inflammatory chemokines in human peripheral blood mononuclear cells. *Indian J. Clin. Biochem*. **29**, 133–138, 10.1007/s12291-013-0345-9 (2014). 24757292 10.1007/s12291-013-0345-9 PMC3990790

[44] Ferat-Osorio, E. et al. The increased expression of TREM-1 on monocytes is associated with infectious and noninfectious inflammatory processes. *J. Surg. Res* **150**, 110–117, 10.1016/j.jss.2007.12.805 (2008). 18656898 10.1016/j.jss.2007.12.805

[45] Xu, P. et al. Microglial TREM-1 receptor mediates neuroinflammatory injury via interaction with SYK in experimental ischemic stroke. *Cell Death Dis.* **10**, 555, 10.1038/s41419-019-1777-9 (2019). 31324751 10.1038/s41419-019-1777-9 PMC6642102

[46] Zhao, T. et al. Inhibition of TREM-1 alleviates neuroinflammation by modulating microglial polarization via SYK/p38MAPK signaling pathway after traumatic brain injury. *Brain Res.* **1834**, 148907, 10.1016/j.brainres.2024.148907 (2024). 38570153 10.1016/j.brainres.2024.148907

[47] Jove, M. et al. Lipids and lipoxidation in human brain aging. Mitochondrial ATP-synthase as a key lipoxidation target. *Redox Biol.* **23**, 101082, 10.1016/j.redox.2018.101082 (2019). 30635167 10.1016/j.redox.2018.101082 PMC6859548

[48] Yin, F. Lipid metabolism and Alzheimer’s disease: clinical evidence, mechanistic link and therapeutic promise. *FEBS J.* **290**, 1420–1453, 10.1111/febs.16344 (2023). 34997690 10.1111/febs.16344 PMC9259766

[49] Gonen, A. & Miller, Y. I. From inert storage to biological activity-in search of identity for oxidized cholesteryl esters. *Front Endocrinol. (Lausanne)* **11**, 602252, 10.3389/fendo.2020.602252 (2020). 33329402 10.3389/fendo.2020.602252 PMC7715012

[50] Zysset, D. et al. TREM-1 links dyslipidemia to inflammation and lipid deposition in atherosclerosis. *Nat. Commun.* **7**, 13151, 10.1038/ncomms13151 (2016). 27762264 10.1038/ncomms13151 PMC5080444

[51] Li, Q. et al. Impaired lipophagy induced-microglial lipid droplets accumulation contributes to the buildup of TREM1 in diabetes-associated cognitive impairment. *Autophagy* **19**, 2639–2656 10.1080/15548627.2023.2213984 (2023). 37204119 10.1080/15548627.2023.2213984 PMC10472854

[52] Hai, Q., & Smith, J. D. Acyl-Coenzyme A: Cholesterol Acyltransferase (ACAT) in cholesterol metabolism: from its discovery to clinical trials and the genomics era. *Metabolites*. **11**, 543, 10.3390/metabo11080543 (2021). 10.3390/metabo11080543 PMC8398989 34436484

[53] Saadane, A. et al. Retinal hypercholesterolemia triggers cholesterol accumulation and esterification in photoreceptor cells. *J. Biol. Chem.* **291**, 20427–20439, 10.1074/jbc.M116.744656 (2016). 27514747 10.1074/jbc.M116.744656 PMC5034040

[54] Chang, C. C., Huh, H. Y., Cadigan, K. M. & Chang, T. Y. Molecular cloning and functional expression of human acyl-coenzyme A:cholesterol acyltransferase cDNA in mutant Chinese hamster ovary cells. *J. Biol. Chem.* **268**, 20747–20755 (1993). 8407899

[55] Chen, L. et al. ACAT1 and metabolism-related pathways are essential for the progression of clear cell renal cell carcinoma (ccRCC), as determined by co-expression network analysis. *Front Oncol.* **9**, 957, 10.3389/fonc.2019.00957 (2019). 31649873 10.3389/fonc.2019.00957 PMC6795108

[56] Shibuya, K. et al. Brain Targeting of Acyl-CoA:Cholesterol O-Acyltransferase-1 Inhibitor K-604 via the intranasal route using a hydroxycarboxylic acid solution. *ACS Omega* **4**, 16943–16955, 10.1021/acsomega.9b02307 (2019). 31646241 10.1021/acsomega.9b02307 PMC6796924

[57] Wang, Y. T. et al. ACAT-1 gene polymorphism is associated with increased susceptibility to coronary artery disease in Chinese Han population: a case-control study. *Oncotarget* **8**, 89055–89063, 10.18632/oncotarget.21649 (2017). 29179498 10.18632/oncotarget.21649 PMC5687668

[58] Sakashita, N. et al. Cholesterol loading in macrophages stimulates formation of ER-derived vesicles with elevated ACAT1 activity. *J. Lipid Res.* **51**, 1263–1272, 10.1194/jlr.M900288 (2010). 20460577 10.1194/jlr.M900288 PMC3035490

[59] Zaidi, S. A. H. et al. Role of acyl-coenzyme A: cholesterol transferase 1 (ACAT1) in retinal neovascularization. *J. Neuroinflammation* **20**, 14, 10.1186/s12974-023-02700-5 (2023). 36691048 10.1186/s12974-023-02700-5 PMC9869542

[60] Inague, A. et al. Oxygen-induced pathological angiogenesis promotes intense lipid synthesis and remodeling in the retina. *iScience* **26**, 106777, 10.1016/j.isci.2023.106777 (2023). 37213234 10.1016/j.isci.2023.106777 PMC10199268

[61] Amato, R. et al. VEGF as a survival factor in ex vivo models of early diabetic retinopathy. *Invest Ophthalmol. Vis. Sci.* **57**, 3066–3076, 10.1167/iovs.16-19285 (2016). 27286364 10.1167/iovs.16-19285

[62] Gerber, H. P. et al. Vascular endothelial growth factor regulates endothelial cell survival through the phosphatidylinositol 3’-kinase/Akt signal transduction pathway. Requirement for Flk-1/KDR activation. *J. Biol. Chem.* **273**, 30336–30343, 10.1074/jbc.273.46.30336 (1998). 9804796 10.1074/jbc.273.46.30336

[63] Shi, X. et al. Inhibition of TNF-alpha reduces laser-induced choroidal neovascularization. *Exp. Eye Res*. **83**, 1325–1334, 10.1016/j.exer.2006.07.007 (2006). 16959248 10.1016/j.exer.2006.07.007

[64] Lei, L. et al. TNF-alpha stimulates the ACAT1 expression in differentiating monocytes to promote the CE-laden cell formation. *J. Lipid Res* **50**, 1057–1067, 10.1194/jlr.M800484-JLR200 (2009). 19189937 10.1194/jlr.M800484-JLR200 PMC2681388

[65] Zhang, C. et al. The role of triggering receptor expressed on myeloid cells-1 (TREM-1) in central nervous system diseases. *Mol. Brain* **15**, 84, 10.1186/s13041-022-00969-w (2022). 36273145 10.1186/s13041-022-00969-w PMC9588203

[66] Bae, Y. S. et al. Macrophages generate reactive oxygen species in response to minimally oxidized low-density lipoprotein: toll-like receptor 4- and spleen tyrosine kinase-dependent activation of NADPH oxidase 2. *Circ. Res* **104**, 210–218 10.1161/CIRCRESAHA.108.181040 (2009). 19096031 10.1161/CIRCRESAHA.108.181040 PMC2720065

[67] Li, H., et al. ACAT1/SOAT1 blockade suppresses LPS-mediated neuroinflammation by modulating the fate of toll-like receptor 4 in microglia. *Int. J. Mol. Sci*. **24**, 5616, 10.3390/ijms24065616 (2023). 10.3390/ijms24065616 PMC10053317 36982689

[68] Choi, S. H. et al. Polyoxygenated cholesterol ester hydroperoxide activates TLR4 and SYK dependent signaling in macrophages. *PLoS One* **8**, e83145, 10.1371/journal.pone.0083145 (2013). 24376657 10.1371/journal.pone.0083145 PMC3871536

[69] Murphy, R. C. & Johnson, K. M. Cholesterol, reactive oxygen species, and the formation of biologically active mediators. *J. Biol. Chem.* **283**, 15521–15525, 10.1074/jbc.R700049200 (2008). 18285330 10.1074/jbc.R700049200 PMC2414298

[70] Pan, P. et al. TREM-1 promoted apoptosis and inhibited autophagy in LPS-treated HK-2 cells through the NF-kappaB pathway. *Int J. Med Sci.* **18**, 8–17, 10.7150/ijms.50893 (2021). 33390769 10.7150/ijms.50893 PMC7738954

